# Supplementary material for: Occupant-centered optimization framework to evaluate and design new dynamic shading typologies
Source: PLoS One. 2020 Apr 24;15(4):e0231554. doi: 10.1371/journal.pone.0231554 (PMC7182202; doi:10.1371/journal.pone.0231554)
Supplement: Attachment — Submitted filename: answer_Reviewer_2.docx [file pone.0231554.s003.docx]

Manuscript PONE-D-19-30597

Answer to Reviewer #2

In this document, the authors address the comments of reviewer 2. The authors would like to thank reviewer 2 for his/her comments aimed at improving the quality of the manuscript.

# General comment:

Reviewer #2: The paper investigated the Genetic Algorithm to optimize external dynamic shading in the USA. The study is rich in content and explanation. Generally, the manuscript is well written and the research novelty is linked to the development of dynamic optimization method. However, there are some comments should be considered to improve the research quality:

Thank you for your encouraging comment. The authors would like to mention that they have proceeded to a reorganization of the paper to better highlight the methodology section independently from the case study. In addition, the aim of the paper was slightly adjusted to reflect the main contribution of the manuscript which is the introduction of a methodology to compare and contrast the performance of different dynamic shading typologies. This change is reflected in the title of the paper that now reads: “Iterative optimization framework to advance the design of new dynamic shading typologies” as well as in the additional section 4.3 that presents results on the usefulness of each degree of freedom for the overall performance of their associated shading systems.

# Detailed comments:

1- The abstract mentioned

"The heating, cooling, artificial lighting and daylight (work plane illuminance) performances are evaluated with Radiance and EnergyPlus based on local weather data."

However, the results mainly focus on daylight analysis while heating, cooling, artificial lighting are superficially covered. A proper balance among different factors should be provided.

The abstract was re-written to integrate the revised focus of the article and the comment of the reviewer. The abstract now reads:

“*Dynamic solar shading has the potential to dramatically reduce the energy consumption in buildings while at the same time providing an improved access to natural daylight for its occupants. Many new typologies of shading systems that have appeared recently, but it is difficult to compare those new systems to existing typologies due to control algorithm being rule-based as opposed to performance driven. Since solar shading is a design problem, there is no single right answer. What is the metric to determine if a system has reached its optimal kinematic design? Shading solution should come from a thorough iterative and comparative process. This paper provides an original and flexible framework for the design and performance optimization of dynamic shading systems based on interpolation and genetic algorithm global minimization. The methodology departs from existing rule-based strategies and applies to existing and to complex shading systems with multiple degree-of-freedom mobility. The strategy for control is centered on meeting comfort targets for work plane illuminance while minimizing the energy needed to operate space. The electric demand for thermal comfort and work plane daylight quantity (illuminance) performances are evaluated with Radiance and EnergyPlus based on local weather data. Applied to a case study of three typologies of dynamic shading, the results of the methodology inform the usefulness and quality of each degree-of-freedom of the kinematic system. The case study exemplifies the iterative benefits of the methodology by providing detailed analytics on the behavior of the shades. Designers of shading systems can use this framework to evaluate their design and compare them to existing shading systems. This allows creativity to be guided so that the building occupants benefits from the innovation in the field.”*

2- The paper should improve its literature with the latest research to support the research decisions, for example:

The study mentioned that the set point of the lighting system is 500 lx without providing an explanation. Also, the study mentioned "The simulated perimeter office is 5 m deep, 4.5 m wide, and 3.2 m high" without providing support from the literature. Including the study below would help to support this claim.

- Luo, Y., Zhang, L., Su, X., Liu, Z., Lian, J., & Luo, Y. (2019). Improved thermal-electrical-optical model and performance assessment of a PV-blind embedded glazing façade system with complex shading effects. Applied Energy, 255, 113896.
- Al-Obaidi, K. M., Munaaim, M. A. C., Ismail, M. A., & Rahman, A. M. A. (2017). Designing an integrated daylighting system for deep-plan spaces in Malaysian low-rise buildings. Solar Energy, 149, 85-101.

Also, the study mentioned about, the algorithm for electric lighting is open-loop; it does not feedback into the daylighting assessment. However, the study does not discuss different feedback systems as open-loop or closed-loop in dynamic shading systems. Including the study below would help to support this claim.

- Al-Masrani, S. M., & Al-Obaidi, K. M. (2019). Dynamic shading systems: A review of design parameters, platforms and evaluation strategies. Automation in construction, 102, 195-216.
- Ayoub, M. (2018). Integrating illuminance and energy evaluations of cellular automata controlled dynamic shading system using new hourly-based metrics. Solar Energy, 170, 336-351.

The authors would like to thank the reviewer for providing the appropriate literature to support the choice of the room size. Those references were included in section 3.1. Similarly, the authors have included the following sentence in Section 2.2 “In that sense, it is similar to the open loop operation system described in [13].” To integrate the comment about the open loop algorithm.

3- The study needs to explain the outdoor daylight and sky conditions in Mercer County, New Jersey, USA that would control the study outcomes.

The authors have added a figure (Figure 3) to explain the outdoor daylight and sky conditions in mercer county throughout the year. Thank you for pointing out this opportunity to drive the point that the reduction of environmental daylight for human comfort is substantial.

4- The study mentioned about 4 sensors as shown in Figure 1 but I couldn't see the results of each sensor.

The four sensors indicated in Figure 1 are the electric lighting sensors. They are used to adjust the lighting level when the daylight does not allow to reach the 500 lux constraint, see Line 296-298 of initial submission for details. The constraint of the optimization is to produce a 500 lux average work plane and therefore a single system was used to compensate the lack of daylight. The daylight quantity produced by the electric lighting system is given as the difference between the average daylight quantity provided and the objective of 500 lux of work plane illuminance. This value can be assessed by looking at figure 9 that represents the average workplane daylight quantity for the three shading systems and for the three orientations of the test room.

5- The study mentioned ""The total run-time of the analysis was 73 minutes on an Intel i7 4 core CPU at 4.20GHz. This total time is decomposed in 21 minutes for the daylighting analysis in Radiance, 42 minutes for the thermal analysis in EnergyPlus and 10 minutes for the optimization in MATLAB."

How could this statement help the outcomes of this research?

The authors believe this information provides the reader unfamiliar with one of the aspects of the study with a benchmark of what each section of the methodology requires in terms of computation. In addition, highlighting the ratio between optimization and simulation allows to point out that the methodology is composed of two parts, simulation and optimization. Once the simulation is performed, the optimization can be run independently for varying values of the constraints chosen by the designer. The following sentence was added in section 5.1 to make this point clearer for the reader:

“*For a given case study, once the simulations have been performed, the optimization can be run for any value of the illuminance constraint without having to perform the simulations again”*

6- Figure 2, model C, it is unclear to understand the model movement and rotation.

The authors have improved the figure to make it more understandable. Namely, the azimuth degree of freedom was clarified and both pictures were made larger.
